# Dashboard Knee: Injury Mechanisms, Diagnostic Challenges, and Treatment Outcomes

**DOI:** 10.7759/cureus.104683

**Published:** 2026-03-04

**Authors:** Christopher Sancilio, Laith Fada, Julian Pulido, Albert D Mousad, Skyler Sorkin, Michael Mastroianni, Greg Jacobs, Frank Mccormick

**Affiliations:** 1 Medical School, Alabama College of Osteopathic Medicine, Dothan, USA; 2 Medical School, University of Illinois at Chicago, Chicago, USA; 3 Orthopaedic Surgery, Geisinger Health System, Wilkes-Barre, USA; 4 Research, Alabama College of Osteopathic Medicine, Dothan, USA; 5 Orthopaedic Surgery, Columbia University, New York, USA; 6 Emergency Medicine, Alabama College of Osteopathic Medicine, Dothan, USA; 7 Department of Orthopedics, Bayonne University, Bayonne, USA

**Keywords:** blunt knee trauma, dashboard knee, frontal collision injury pattern, knee arthroscopy, posterior cruciate ligament injury

## Abstract

Introduction: Frontal motor vehicle collisions (MVCs) are the most common crash configuration and are a well-recognized mechanism for posterior cruciate ligament (PCL) injury through direct dashboard impact. These "dashboard knee" injuries may initially present with subtle clinical findings yet progress to persistent pain, instability, and functional limitation if not appropriately identified and managed. Despite the recognized mechanism, early recovery patterns and short-term outcomes following surgical versus conservative management remain incompletely characterized.

Methods: A retrospective case series was conducted of patients presenting to a single orthopedic surgeon with magnetic resonance imaging (MRI)-confirmed PCL injury following frontal MVC between June 2022 and January 2024. Ten patients met the inclusion criteria. Five patients underwent arthroscopic PCL reconstruction with treatment of associated intra-articular pathology when indicated, and five patients were managed conservatively with structured physiotherapy. Patient-reported pain scores, measured using a six-point ordinal scale, were collected at 4, 8, and 12 weeks following treatment initiation to evaluate recovery trends. Descriptive and comparative analyses were performed.

Results: Nine of 10 patients demonstrated progressive improvement in pain scores over the 12-week follow-up period. One patient in the conservatively managed group experienced worsening symptoms. Patients treated surgically demonstrated lower cumulative pain scores, with an average total score of 8 out of 18, compared with 13 out of 18 in the conservative group. Conservatively treated patients also reported longer symptom duration, increased stiffness, and persistent swelling.

Conclusion: Patients with PCL injuries resulting from dashboard mechanisms demonstrated improvement with both surgical and conservative management; however, surgical intervention was associated with greater early pain reduction in this cohort. These findings underscore the importance of early recognition of PCL injury following frontal MVC and suggest that surgical reconstruction may provide improved short-term symptom relief in appropriately selected patients. Larger studies are needed to further define optimal management strategies.

## Introduction

The phenomenon of the "dashboard knee" represents a distinctive and clinically significant injury pattern within orthopedic trauma, most commonly associated with posterior cruciate ligament (PCL) disruption following frontal motor vehicle collisions (MVCs). Frontal collisions account for approximately 50%-60% of all MVCs, making them the most frequent crash configuration associated with lower extremity injury [1]. The injury mechanism involves a posteriorly directed force applied to the proximal tibia while the knee is flexed, typically during dashboard impact. This force translates the tibia posteriorly relative to the femur, resulting in stretching, partial tearing, or complete rupture of the PCL, which is the most commonly injured ligament in dashboard-related trauma. Concomitant injuries to the menisci, collateral ligaments, and articular cartilage may also occur, contributing to greater joint instability and long-term morbidity [2,3].

Although the mechanism of injury is well recognized, the true incidence and burden of dashboard-related knee injuries remain underreported. The challenge lies in the lack of obvious signs of instability or gross swelling in the acute setting, as well as potentially normal radiographic findings. These factors contribute to diagnostic delays, especially in polytrauma settings, where immediate attention is directed toward life-threatening injuries [4]. Magnetic resonance imaging (MRI) remains the gold standard for detecting these injuries. However, MRI is not routinely used early in the post-trauma period for this injury pattern, particularly in emergency care environments [5]. These diagnostic limitations may result in bone and ligament damage that remains clinically silent, increasing the risk of pain, functional decline, and degenerative changes [6].

Management strategies for dashboard knee injuries vary depending on injury severity and associated ligament involvement. Conservative treatment, including physiotherapy, quadriceps strengthening, and bracing, remains appropriate for low-grade isolated PCL injuries. However, the optimal management of high-grade or multiligament injuries remains debated. While several studies suggest improved stability and functional outcomes with surgical reconstruction in selected patients, other reports demonstrate satisfactory outcomes with nonoperative treatment, highlighting the need for further investigation into treatment-specific recovery patterns [6-8].

Although prior literature has described the injury mechanism and treatment options, there remains limited understanding of short-term recovery patterns and comparative outcomes between conservative and surgical management, specifically in patients with dashboard-related knee injuries following frontal MVCs. In particular, few studies have evaluated early patient-reported outcomes such as pain, stiffness, and swelling during the initial recovery period.

Therefore, this study aims to provide a retrospective review of patients with confirmed dashboard-related knee injuries following frontal MVCs and to compare short-term outcomes between surgical and conservative management. Recovery patterns were evaluated through self-reported pain scales and clinical assessments over a 12-week follow-up period. By examining early treatment outcomes, this study seeks to clarify recovery trajectories, inform clinical decision-making, and improve early diagnostic and management strategies for this underrecognized injury pattern.

## Materials and methods

This retrospective case series was conducted at a single orthopedic specialty practice and affiliated outpatient surgical center in Florida, United States. The study evaluated patients who sustained posterior cruciate ligament (PCL) injuries resulting from dashboard-related knee trauma during frontal motor vehicle collisions (MVCs) and were evaluated and treated by a single board-certified orthopedic surgeon between June 2022 and January 2024. Dashboard-related injury was defined as a direct anterior force to the proximal tibia resulting from contact with the vehicle dashboard at the time of impact.

Patients were identified through retrospective review of electronic medical records, operative reports, and imaging studies. Inclusion criteria consisted of: (1) documented history of direct anterior knee impact during a frontal MVC consistent with a dashboard mechanism, (2) clinical findings suggestive of PCL injury, and (3) magnetic resonance imaging (MRI) confirmation of partial or complete PCL tear. Associated intra-articular injuries, including meniscal tears or chondral lesions, were included if present in conjunction with PCL injury. All patients were required to have a minimum follow-up duration of 12 weeks.

Patients were excluded if they sustained concurrent fractures, had prior knee surgery, inflammatory arthropathy, radiographic osteoarthritis, or pre-existing knee pathology unrelated to the traumatic event that could confound outcome assessment. Patients with concomitant anterior cruciate ligament (ACL) injuries requiring separate reconstruction were excluded to maintain a more homogeneous study population focused on PCL injury patterns.

All patients underwent standardized orthopedic evaluation at initial presentation by the treating orthopedic surgeon. Clinical assessment included inspection for swelling, effusion, ecchymosis, and deformity; palpation for joint line tenderness; assessment of active and passive range of motion; and ligamentous stability testing, including posterior drawer test, anterior drawer test, and varus and valgus stress testing. Neurovascular examination was performed and documented in all cases. MRI was ordered by the treating orthopedic surgeon in patients with clinical suspicion for ligamentous injury to confirm the diagnosis, assess injury severity, and guide treatment planning.

Patients were categorized into surgical and conservative management groups based on symptom severity, degree of instability, functional limitation, and imaging findings. The surgical group underwent arthroscopic PCL reconstruction or repair, with or without treatment of associated intra-articular pathology, at an affiliated surgical center. Arthroscopic intervention included diagnostic arthroscopy followed by reconstruction of the PCL using standard graft fixation techniques and concurrent management of associated injuries such as meniscal repair, partial meniscectomy, or chondroplasty when indicated.

The conservative management group was treated with a structured nonoperative protocol consisting of physiotherapy emphasizing quadriceps strengthening, restoration of range of motion, and proprioceptive training. Patients were advised to modify activities and avoid high-impact loading during early recovery. Nonsteroidal anti-inflammatory medications were prescribed as needed.

Patient-reported outcomes were assessed using a study-specific Lower Limb Questionnaire (LLQ), designed to capture symptoms relevant to PCL injury, including pain, stiffness, swelling, and functional limitation. Although the LLQ is not a previously validated instrument, it was developed to assess clinically relevant symptoms observed in patients with PCL injury and was administered consistently at each follow-up visit. Pain severity was measured using a six-point ordinal scale ranging from 1 (no pain) to 6 (severe pain), allowing assessment of symptom progression over time.

Outcome measures were recorded at baseline and at 4, 8, and 12 weeks following treatment initiation or surgical intervention.

Descriptive and comparative statistical analyses were performed using SPSS software (IBM Corp., Armonk, NY). Continuous variables were reported as means with standard deviations, and categorical variables were reported as frequencies and percentages. Comparisons between surgical and conservative groups were performed using Student’s t-test for continuous variables and chi-square test for categorical variables. Changes in pain scores over time were analyzed using repeated-measures analysis of variance (ANOVA). Statistical significance was defined as p < 0.05.

Due to the retrospective nature of the study and the limited number of eligible patients during the study period, a formal sample size calculation was not performed. All eligible patients meeting the inclusion criteria were included.

## Results

A total of ten patients were included, with five undergoing operative reconstruction and five managed nonoperatively. Due to the small sample size, formal statistical comparison was not performed. Outcomes are presented descriptively as mean ± standard deviation.

At one month, operative and nonoperative groups demonstrated similar baseline symptom severity. Mean stiffness was 3.37 ± 0.75 in operative patients and 3.50 ± 1.52 in nonoperative patients. Swelling averaged 3.20 ± 0.45 and 3.20 ± 0.84, respectively. Pain with walking was 4.00 ± 1.00 in operative patients and 4.40 ± 0.89 in nonoperative patients. Pain with stair climbing was 4.60 ± 0.89 and 4.40 ± 0.89, respectively. Pain while lying in bed was identical between groups at 4.40, with standard deviations of 0.55 and 0.89 in operative and nonoperative patients, respectively.

At two months, improvement was observed in both groups, with greater mean reductions in operative patients. Mean stiffness improved to 2.50 ± 0.84 in operative patients compared with 3.33 ± 0.52 in nonoperative patients. Swelling decreased to 2.40 ± 0.89 following surgery but remained elevated at 3.40 ± 0.55 in nonoperative patients. Pain with walking improved to 3.00 ± 0.71 in operative patients but remained unchanged at 4.40 ± 0.89 in nonoperative patients. Pain with stair climbing improved to 3.60 ± 0.89 in operative patients compared with 4.20 ± 0.84 in nonoperative patients. Pain while lying in bed decreased to 2.80 ± 1.10 following surgery and to 4.00 ± 1.00 in nonoperative patients.

At three months, continued improvement was observed in both groups. Operative patients demonstrated lower mean stiffness (2.23 ± 0.75) compared with nonoperative patients (3.10 ± 0.68). Swelling improved to 2.00 ± 0.71 in operative patients compared with 3.00 ± 0.71 in nonoperative patients. Pain with walking decreased to 2.40 ± 1.14 following surgery and to 3.60 ± 1.14 in nonoperative patients. Pain with stair climbing improved to 2.80 ± 1.30 in operative patients compared with 4.20 ± 1.10 in nonoperative patients. Pain while lying in bed was 3.00 ± 1.22 in operative patients and 4.00 ± 1.00 in nonoperative patients.

Overall, both operative and nonoperative management were associated with symptom improvement over time. Greater mean reductions in stiffness, swelling, and pain were observed in operative patients; however, statistical significance could not be determined due to the limited sample size.

**Figure 1:**
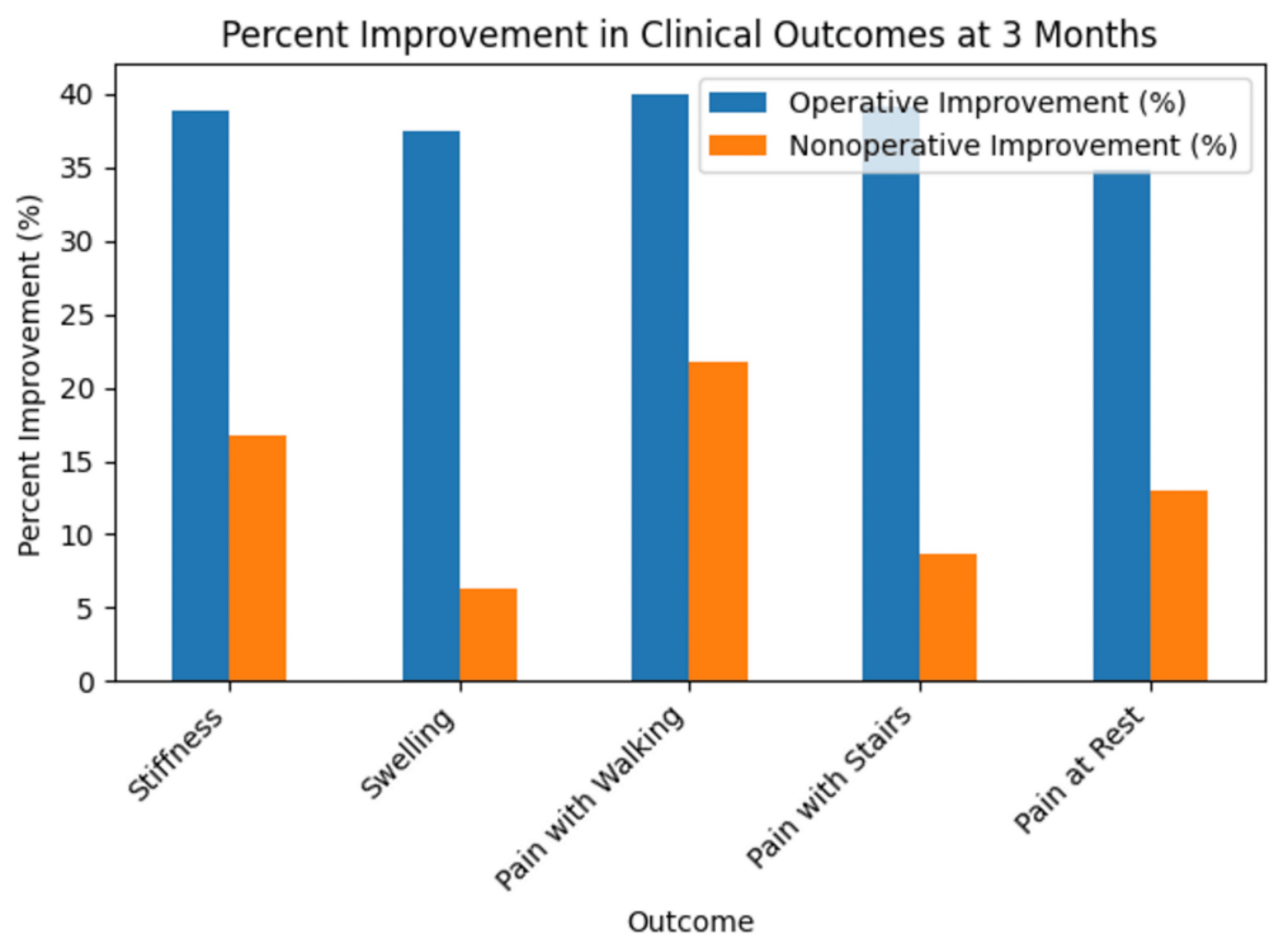
Operative and nonoperative comparison of results at three months.

These findings are illustrated in Figure 1. 

## Discussion

Dashboard knee injuries, caused by anterior impact of the proximal tibia against the femur during frontal motor vehicle collisions, remain underreported in the orthopedic literature. The true incidence is likely underestimated due to subtle early clinical findings, including minimal swelling, preserved range of motion, or absence of radiographic abnormalities on initial evaluation [7]. Although MRI remains the gold standard for detecting soft tissue injuries, including posterior cruciate ligament (PCL) disruption, its use in the acute trauma setting is inconsistent, which may contribute to delayed diagnosis and treatment [8].

In this retrospective cohort, both operative and nonoperative management were associated with improvement in stiffness, swelling, and pain over the three-month follow-up period. Operative patients demonstrated greater mean improvement across multiple outcome domains, including stiffness, swelling, and pain with ambulation and stair climbing. Surgical reconstruction restores posterior stability by reestablishing the native biomechanical restraint to posterior tibial translation, thereby improving tibiofemoral kinematics and reducing abnormal joint loading [9]. In addition, arthroscopically assisted PCL reconstruction allows direct visualization of intra-articular structures, enabling identification and treatment of associated pathology, including meniscal and chondral injuries, during the same procedure.

**Figure 2:**
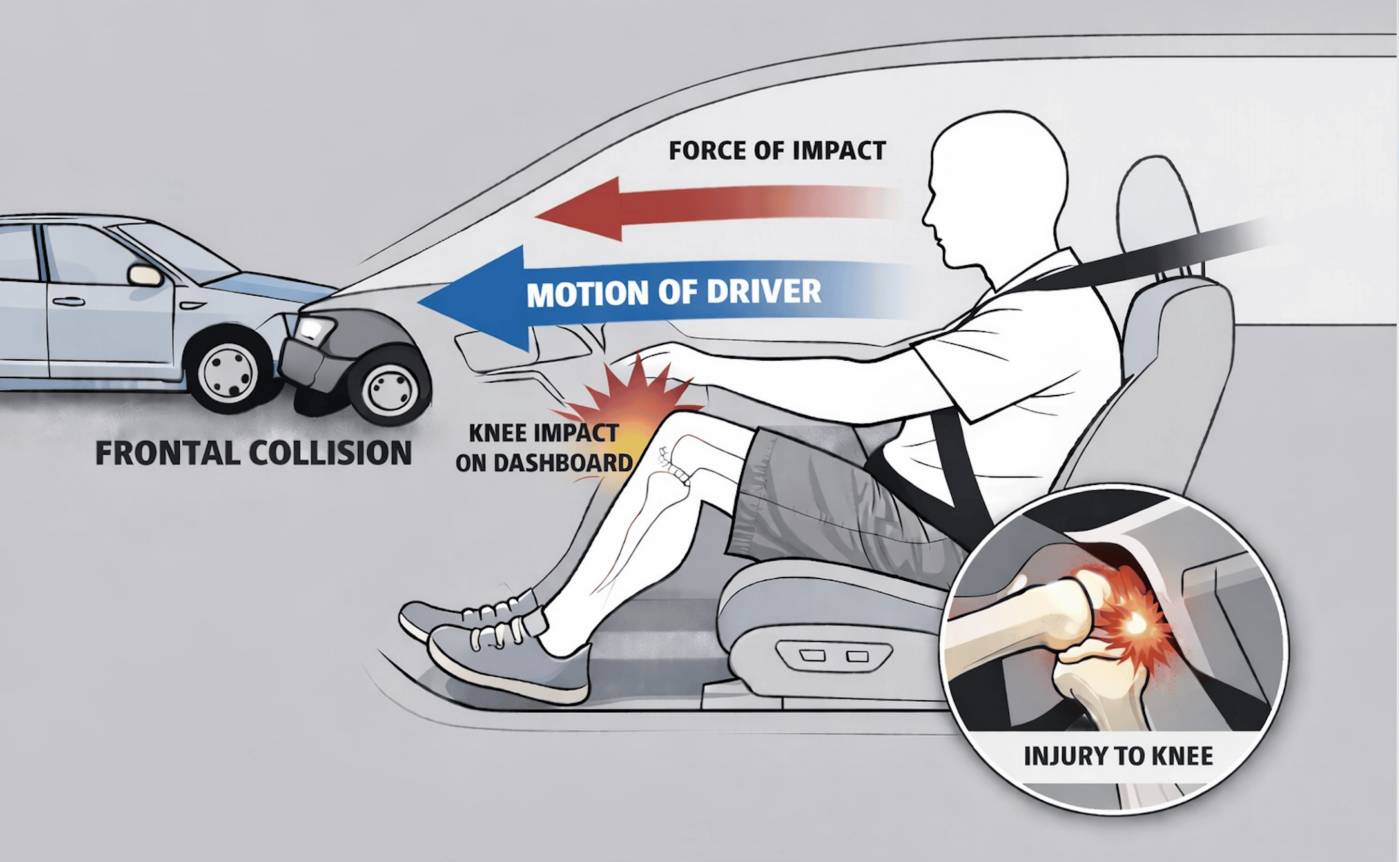
Mechanism of injuries in frontal motor vehicle collisions (dashboard knee). This image was created by the authors for visual purposes.

Nonoperative treatment remains an acceptable approach for low-grade PCL injuries, particularly in younger or less active patients with strong quadriceps recruitment capable of dynamic stabilization. However, outcomes tend to be less predictable in complete tears or high-demand populations, where persistent posterior sag and instability frequently compromise performance and comfort. Several studies have demonstrated that prolonged conservative treatment in high-grade injuries predisposes patients to early degenerative changes, emphasizing the importance of timely surgical management [10]. As illustrated in Figure 2, the mechanism of dashboard knee typically involves a sudden posteriorly directed force applied to the proximal tibia with the knee in flexion, producing joint compression and shear stresses that frequently injure the PCL.

**Figure 3:**
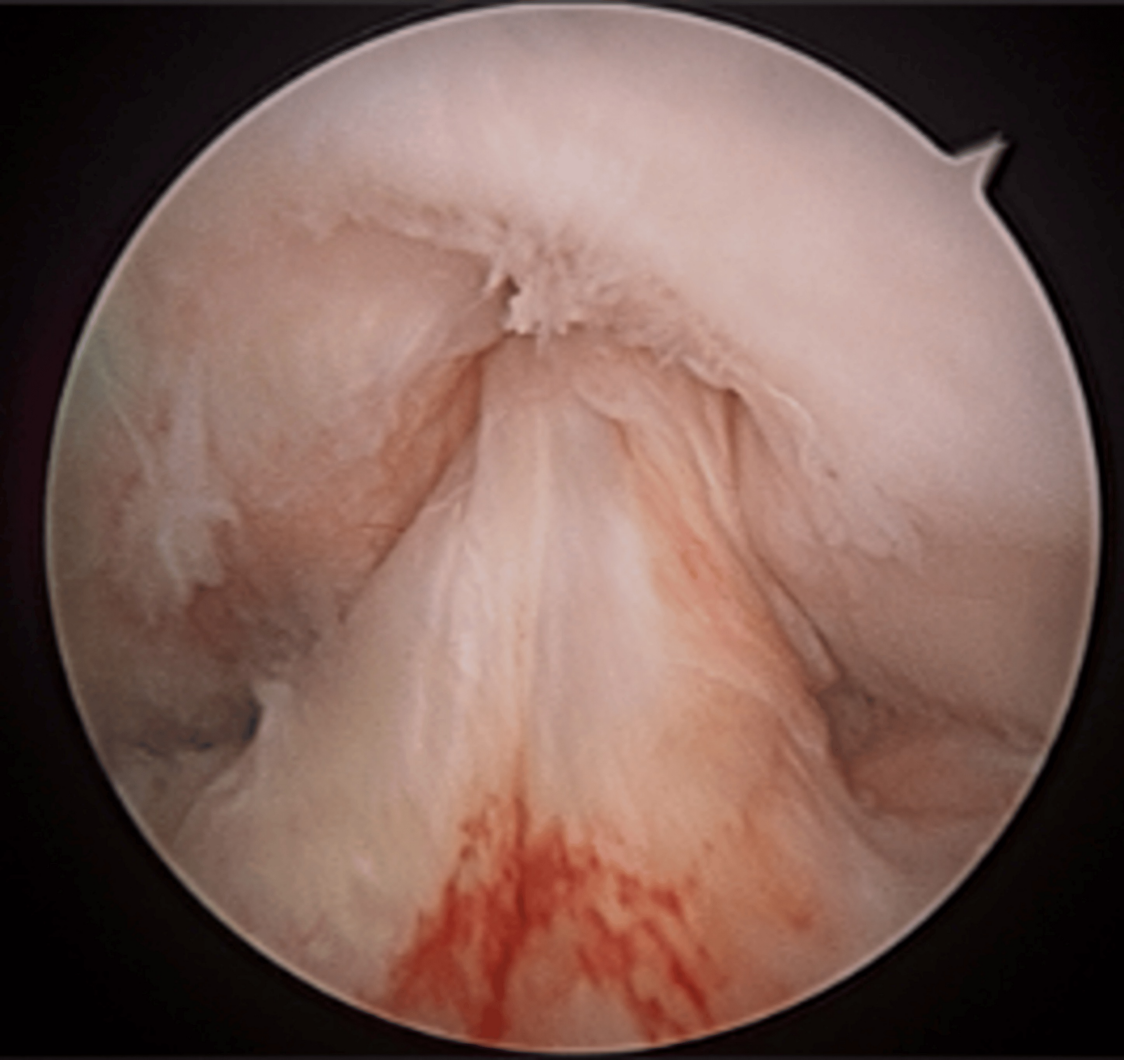
Arthroscopic view of the anterior cruciate ligament (ACL) demonstrating partial fiber inflammation without pathological tearing of the ligament following dashboard knee trauma. Early surgical visualization allows for accurate assessment and timely stabilization.

Arthroscopy of the knee joint not only facilitates visualization and confirmation of PCL injury but also enables identification and management of associated intra-articular pathology, such as meniscal root tears, chondral lesions, and capsular disruptions [11] (Figure 3). Arthroscopically assisted PCL reconstruction techniques allow precise graft placement and tensioning while minimizing surgical morbidity associated with traditional open approaches [12]. This approach provides a comprehensive view of the tibiofemoral and patellofemoral compartments, ensuring accurate restoration of the ligament’s anatomic properties. Moreover, it allows simultaneous treatment of concomitant injuries frequently seen in dashboard mechanisms, including meniscocapsular separations and posterior tibial bone contusions resulting from posterior tibial translation [13]. In contrast, extra-articular reconstruction techniques may restore gross stability but fail to correct abnormal knee kinematics or address secondary intra-articular damage. Arthroscopic reconstruction, therefore, offers a more integrated approach by restoring posterior stability, preserving native joint architecture, and optimizing early functional recovery in dashboard-related PCL injuries [14].

Early recognition and appropriate management of dashboard-related PCL injuries remain critical. Delayed diagnosis may lead to chronic posterior instability, progressive cartilage degeneration, and long-term functional impairment [15]. Persistent abnormal tibiofemoral mechanics may increase the risk of degenerative joint disease, particularly in younger and more active individuals [16].

Several limitations must be considered when interpreting these findings. The small sample size limits statistical analysis and reduces the ability to draw definitive conclusions regarding comparative effectiveness. The retrospective design introduces potential selection bias, as treatment allocation was determined by surgeon preference rather than randomization. Patients selected for operative intervention may have differed in injury severity, activity level, or clinical presentation compared with those managed conservatively. Additionally, the short follow-up period restricts assessment of long-term outcomes, including return to baseline function, recurrent instability, and progression to degenerative joint disease. This study was conducted at a single center, which may limit generalizability. Furthermore, outcome measures were based on patient-reported symptom scales that have not been formally validated for this specific injury pattern. Variability in rehabilitation protocols and patient adherence may also have influenced recovery trajectories.

Given these limitations, the findings of this study should be interpreted as descriptive observations of short-term recovery patterns. Larger, prospective, randomized studies with long-term follow-up are necessary to better define the optimal management of dashboard-related PCL injuries and to determine the comparative effectiveness of operative and nonoperative treatment strategies.

## Conclusions

In this retrospective case series, both operative and nonoperative management of dashboard knee injuries were associated with improvement in pain and functional symptoms over the early recovery period. Patients who underwent surgical reconstruction demonstrated greater mean improvement across multiple outcome measures, including stiffness, swelling, and pain; however, statistical comparison was not performed due to the small sample size. These findings suggest that surgical management may be beneficial in selected patients, particularly those with persistent instability or higher functional demands. Early recognition of posterior cruciate ligament injury following dashboard mechanisms remains essential to guide appropriate treatment and optimize recovery. Larger prospective studies with longer follow-up are needed to better define the role of surgical intervention in these injuries.
